# Comparison of User-Directed and Automatic Mapping of the Planned Isocenter to Treatment Space for Prostate IGRT

**DOI:** 10.1155/2013/892152

**Published:** 2013-11-21

**Authors:** Zijie Xu, Ronald Chen, Andrew Wang, Andrea Kress, Mark Foskey, An Qin, Timothy Cullip, Gregg Tracton, Sha Chang, Joel Tepper, Di Yan, Edward Chaney

**Affiliations:** ^1^Department of Radiation Oncology, CB 7512, University of North Carolina, Chapel Hill, NC 27599 7512, USA; ^2^Morphormics, Inc., 240 Leigh Farm Road, Durham, NC 27707, USA; ^3^Department of Radiation Oncology, William Beaumont Hospital, Royal Oak, MI, USA

## Abstract

Image-guided radiotherapy (IGRT), adaptive radiotherapy (ART), and online reoptimization rely on accurate mapping of the radiation beam isocenter(s) from planning to treatment space. This mapping involves rigid and/or nonrigid registration of planning (pCT) and intratreatment (tCT) CT images. The purpose of this study was to retrospectively compare a fully automatic approach, including a non-rigid step, against a user-directed rigid method implemented in a clinical IGRT protocol for prostate cancer. Isocenters resulting from automatic and clinical mappings were compared to reference isocenters carefully determined in each tCT. Comparison was based on displacements from the reference isocenters and prostate dose-volume histograms (DVHs). Ten patients with a total of 243 tCTs were investigated. Fully automatic registration was found to be as accurate as the clinical protocol but more precise for all patients. The average of the unsigned *x, y,* and *z* offsets and the standard deviations (**σ**) of the signed offsets computed over all images were (avg. ±  **σ** (mm)): 1.1 ± 1.4, 1.8 ± 2.3, 2.5 ± 3.5 for the clinical protocol and 0.6 ± 0.8, 1.1 ± 1.5 and 1.1 ± 1.4 for the automatic method. No failures or outliers from automatic mapping were observed, while 8 outliers occurred for the clinical protocol.

## 1. Introduction

**Figure 1:**
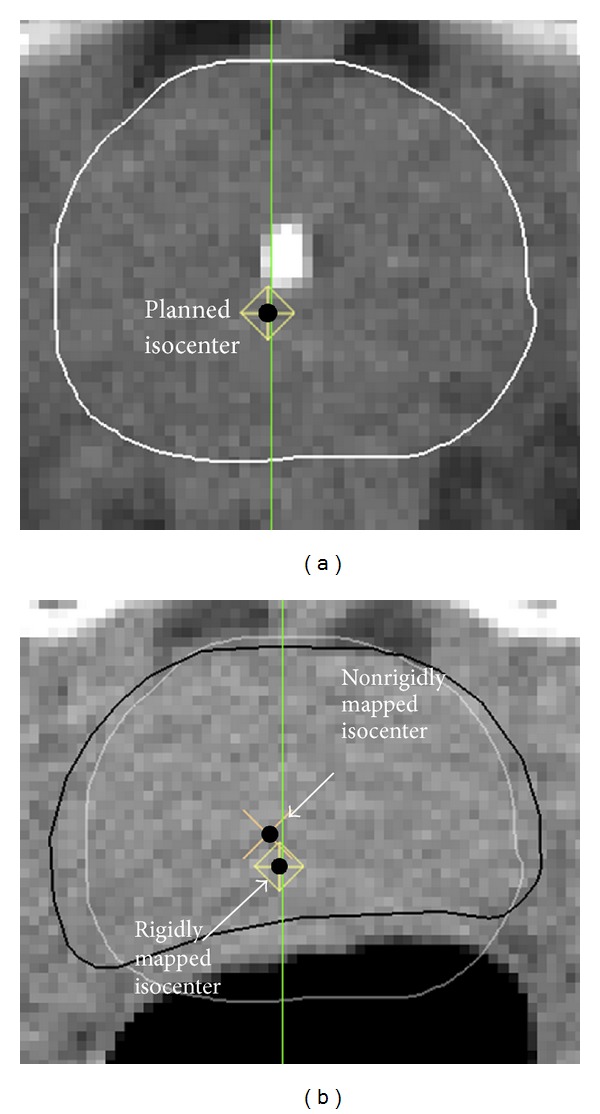
(a) Axial slice from the pCT showing planning prostate (white) and isocenter (black). (b) Corresponding slice from the tCT showing the prostate (black) segmented by automatic nonrigid model deformation. The rigidly mapped isocenter comes from translating the planning prostate (dim white) to the tCT. The nonrigidly mapped isocenter comes from applying the deformation matrix resulting from autosegmentation to the planned isocenter.

Image-guided radiotherapy (IGRT) [1], off-line adaptive radiotherapy (ART) [2], and online reoptimization [3] involve pretreatment imaging, taken here to be CT imaging. A procedure held in common by all three methods is registration of the planning (pCT) and treatment (tCT) images to map the planned isocenter to treatment space. Accuracy and precision of this step are important for delivering an accumulated dose distribution that closely matches the treatment plan. Mapping methods involve at least rigid registration. Ideally a nonrigid step would be included to account for differences in organ shape between planning and treatment times (Figure 1). The composite of the rigid, and possibly nonrigid, matrices is then used to map the planned isocenter to the tCT. 

Quantitative evaluation of isocenter mapping methods is muddled by the lack of gold standards [4]. In the absence of standards, this work retrospectively compared a fully automatic method against a user-assisted procedure used in a clinical protocol for IGRT for prostate cancer. The study focused on tCTs from a conventional diagnostic scanner due to the availability of clinical data. However, the approach applies to kilovoltage cone-beam CT (CBCT) images as illustrated later.

## 2. Methods

### 2.1. Clinical Protocol

The clinical protocol (Table 1) was practiced over the period 2005–2008 at the University of North Carolina (UNC) as part of the routine workflow during evaluation of a CT-on-rails system [5] (Primatom, Siemens Medical Solutions, Concord, CA). The Primatom system consists of a conventional CT scanner, a set of rails between the scanner and the linac, and a dual-purpose imaging/treatment couch that moves along the rails. Patients under the IGRT protocol were imaged before treatment, and the couch was rolled from the CT scanner into the image-guided treatment position. The fixed geometry between the scanner and linac allows cross calibration of the patient laser alignment systems, and the rails facilitate moving the couch from the scanner into treatment position with minimal time delay or mechanical disturbance of patient geometry.

**Figure 2:**
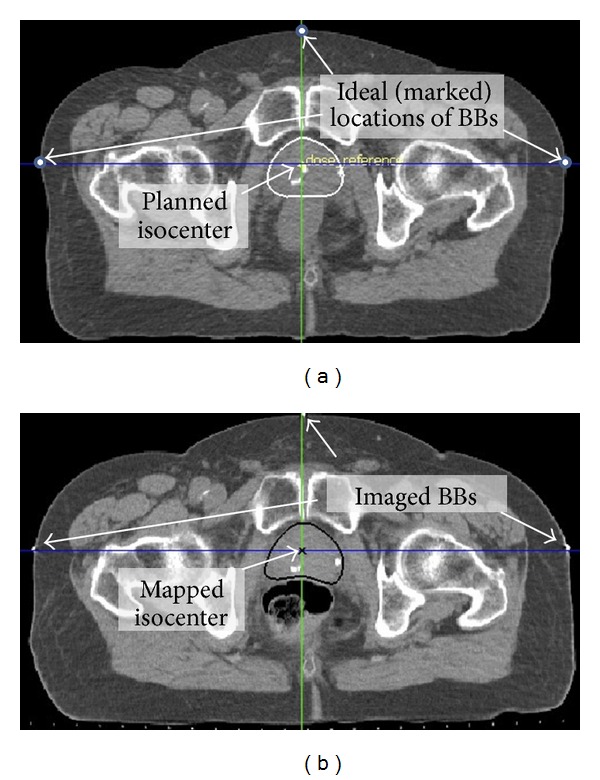
(a) Axial slice from the pCT showing planned isocenter inside the prostate (white contour) and the marked locations of BBs. (b) Corresponding slice from the tCT showing the autosegmented prostate (black), nonrigidly mapped isocenter, and imaged BBs.

All patients underwent simulation using a Philips AcQSim CT system [6] (Philips Healthcare, Andover, MA). The planning isocenter was localized during simulation, and the anterior and lateral laser crosshairs were tattooed according to standard practice. Treatment planning and calculation of hypothetically delivered dose were accomplished with PlanUNC [7, 8]. (PLanUNC is a set of modular software tools for external beam treatment planning and dose calculation development at UNC.) During planning the dosimetrist placed the computer crosshairs at the planned isocenter and then marked reference points in the pCT at the intersections of the computer crosshairs with the skin (Figure 2). These reference points defined the geometrically correct positions of the anterior and lateral laser crosshairs based on patient geometry at planning time. Before acquisition of each tCT, the patient was positioned by aligning the tattoos on the patient's skin with the CT laser crosshairs. After alignment, steel BBs were taped to the skin at the center of each laser's crosshairs (AP, R&L lat). Assuming accurate laser calibration, this placement allows the treatment isocenter for the initial patient position to be inferred from the imaged BBs. Immediately after imaging, the tCT was imported to the planning system and the physicist defined the BB centers via point and click on a computer display screen. The planning system then automatically registered the tCT and pCT by matching the BBs in the tCT with the ideal locations marked in the pCT. The algorithm minimized ∑(distance between corresponding points)^2^. The final step was interactive rigid registration of the prostate ROIs using the planning contours for reference. Table tilt around the lateral axis and rotation around the craniocaudal axis were not allowed in the final two steps because rotational errors were not handled during patient setup. Since the prostate was not contoured in the tCT, the final step relied on human judgment to match the pCT intensities and contours with the tCT intensity patterns in the prostate ROI. Table displacements were computed by comparing the coordinates of the mapped isocenter after BB registration with the coordinates after interactive prostate-based registration. The displacements were used to reposition the treatment table. Assuming accurate registration and repositioning, and no changes in patient anatomy between tCT imaging and treatment times, this procedure registered the mapped planned isocenter with the treatment isocenter. In typical practice of IGRT, displacements are implemented only when they exceed a predefined threshold. In the UNC protocol, the threshold was 3 mm along a given axis. However, for comparison purposes this study assumed that the displacements were applied without error regardless of magnitude. 

### 2.2. Automatic Mapping Method

Automapping (Table 1) was performed using a beta version of ARTSuite (Morphormics, Inc. Chapel Hill, NC) installed at UNC. (Effective July 16, 2012, Morphormics, Inc. became a wholly owned subsidiary of Accuray, Inc., Sunnyvale, CA.) ARTSuite is a DICOM-RT compliant software system developed to support IGRT, ART, and online reoptimization with tools for autosegmentation of pCTs and tCTs, nonrigid mapping between treatment and planning spaces, and dose accumulation and analysis.

**Figure 3:**
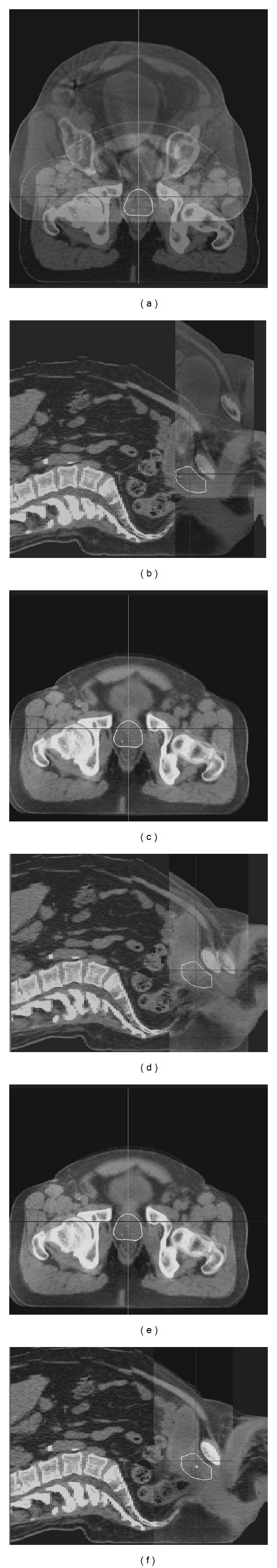
Multiscale registration of a pCT with a CBCT. The planning prostate segmentation is white. ((a), (b)) Blended axial and sagittal slices of unregistered images. ((c), (d)) Axial and sagittal slices of images registered via Step (1). ((e), (f)) Axial and sagittal slices of images registered via mutual information in the prostate ROI (Step (3)). In this example, there is little difference between the second and third steps.

Automapping (Figure 3) involved three major steps: (1) multiscale rigid registration of the previously segmented pCT to the tCT, (2) model-based autosegmentation of the prostate in the tCT [9, 10], and (3) nonrigid mapping of the planning isocenter to the tCT using a transformation that included the rigid registration matrix and a nonstatistical, nonrigid diffeomorphism determined from correspondences between the pCT and tCT prostate models. 

### 2.3. Patient Data

Ten patients with a total of 243 tCTs were investigated. All pCTs were acquired with contrast in the bladder, and one patient had contrast in the bladder and rectum. The pixel dimensions in the axial plane were 1 mm × 1 mm, and the slice thickness was 3 mm for pCTs and tCTs. 

All patients were treated with step-and-shoot IMRT using an anterior and six oblique fields to ~75 Gy prescribed to a point or isodose curve. Treatment typically included a boost starting ~50 Gy to reach the final dose. Margins of 5 mm and 3 mm were applied to the planning target volume for the initial and boost plans, respectively. 

#### 2.3.1. Model Fitting to Prostate Contours

 This study assumed that the manually drawn prostate contours in the pCT were true at treatment time. To facilitate model-based segmentation of the tCTs as described below, a prostate model was fit to each set of planning contours, using an approach based on prostate shape statistics described by Merck et al. [11]. This process yielded a custom prostate atlas for each patient that was used during registration and segmentation of tCTs. Contours were represented as many short line segments joined together. This representation caused small differences between a contour and a model at sharp vertices where two line segments joined. These differences were less than 0.2 mm per contour on average and were caused by smoothness constraints that forced the model to have continuous curvature. 

#### 2.3.2. Multiscale Rigid Registration

**Figure 4:**
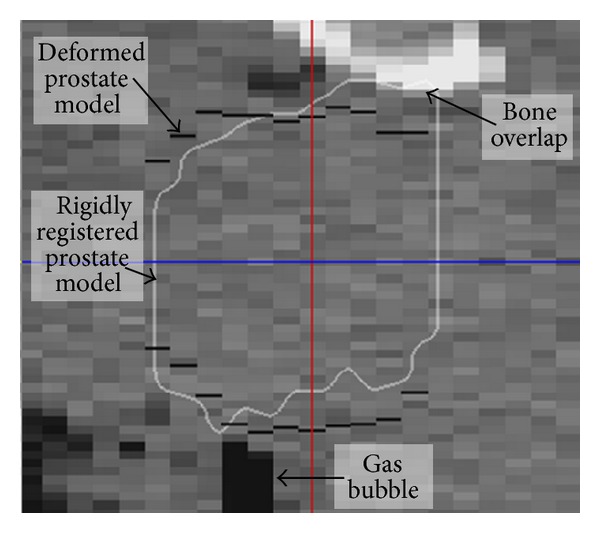
Midsagittal slice from a tCT illustrating how prostate rotation is treated as a deformation. The white outline is the atlas prostate after automatic registration to the tCT. The black dashes are the deformed atlas in the tCT.

 The overall rigid registration approach has similarities with that of Court and Dong [12] and Smitsmans et al. [13], discussed later in Section 3. To minimize compute time, registration progresses from coarse to fine scale in three steps. The output of each step serves as a prior for the following step. Step (1) aligns the skin bounding boxes and registers the images by sliding the image data in the bounding box in the pCT along the box for the tCT. The registration algorithm computes the voxel count per slice in a predefined intensity window and aligns the images by finding the best match between graphs of voxel count versus slice position for each image pair. A bone window performs best but can fail when a “bright” contrast medium is used for the pCT. In such cases, Step (1) is ignored and the algorithm starts over at Step (2), which optimizes global mutual information (MI) using a gradient descent approach [14]. To avoid convergence to a distant optimum, the algorithm is run multiple times with different starting points. The result with the best score over all runs is selected as the output. Step (3) is similar to Step (2) but with MI computed over an ROI defined by the atlas prostate model. As in the clinical procedure, rotations during automatic registration were not allowed explicitly. However, the segmentation step treated rotation as a nonrigid deformation in images with adequate intensity information. In Figure 4, for example, the rigidly registered prostate model overlaps pubic bone anteriorly, causing the model to deform in a manner that avoids overlap with both the bone and the gas bubble in the rectum.

#### 2.3.3. Model-Based Autosegmentation

**Figure 5:**
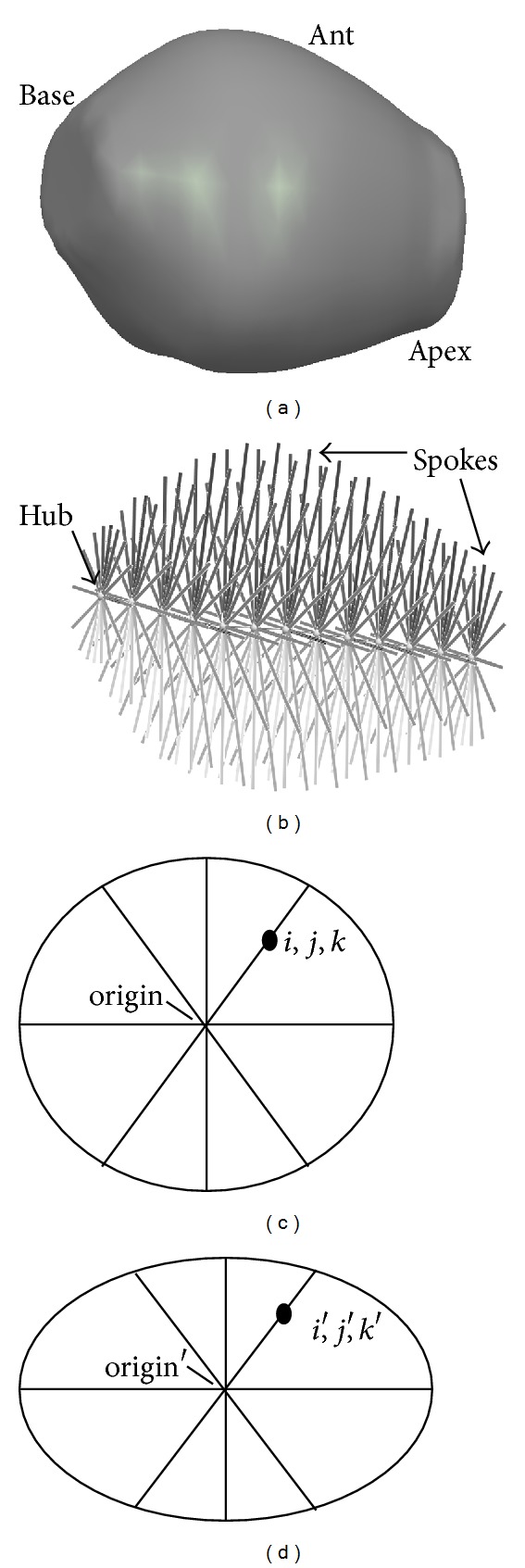
(a) Lateral oblique view of the surface of a prostate model. (b) Internal tubular skeleton showing a chain of 13 atoms, each comprising a hub and 16 spokes that touch the prostate surface. (c) and (d) Illustration of corresponding points in reference and deformed models. (c) Reference model with point at *i*, *j*, *k*. (d) Deformed model with corresponding point at *i*′, *j*′, *k*′. The points are on the same spoke and they have the same fractional distance from the origin (atom hub).

 The model used to represent the prostate consists of a chain of so-called medial atoms (Figure 5). A full collection of atoms for an organ is called a medial-representation (m-rep) [9]. The chain configuration is well suited for objects that are more or less tube-like, including objects with closed ends. Each prostate atom has a hub and 16 spokes radiating to the organ surface. Additional hubs and spokes can be interpolated as needed. The skeletal framework serves as an organ-relative coordinate system with a formalism for converting back and forth between model and image coordinates [9, 15]. After deformation of the starting model in a target image, corresponding positions are defined by pairing points in the starting and deformed models that have the same model-relative coordinates (Figure 5). The nonrigid transformation matrix can be specified in image coordinates in terms of a standard displacement vector field [16], where each vector originates on a voxel in the reference image and terminates on its postdeformation position in the target image. In contrast to voxel-scale deformations, the model approach is statistical at the scale of an organ but nonstatistical at voxel scale, eliminating small-scale artifacts [16]. 

Segmentation of the prostate in the tCT is necessary to determine corresponding points in the pCT and tCT. The algorithm transfers the atlas model to the tCT using the rigid registration matrix, and autosegmentation proceeds in a statistical framework based on Bayes' theorem [9]. A conjugate gradient algorithm seeks to find the optimal model ${\underline{M}}_{\text{opt}}$ such that

(1) $$\begin{array}{c} {\underline{M}}_{\text{opt}}=\underset{\underline{M}\in s}{\text{argmax}}\left[\log p\left(\underline{M}\right)+\log p\left(\underline{I}\;|\;\underline{M}\right)\right], \end{array}$$

 where $\underline{M}$ is the currently deformed model in the trained shape space *s* [11],  $\underline{I}$ is the target image intensity pattern relative to $\underline{M},\;\;p(\underline{M})$ is the probability of $\underline{M}$ (geometric typicality), and  $p\left(\underline{I}|\underline{M}\right)$ is the probability of $\underline{I}$ given $\underline{M}$ (image match [17]).

#### 2.3.4. Mapping the Isocenter

 The rationale for using a model to map the isocenter stems from several considerations: (1) during planning the isocenter is positioned relative to the prostate; (2) a point in an image can be more accurately found by relying on regional image features that are correlated spatially with the point rather than using local information near the point itself [18]; and (3) the trainable models used in this study provide a means for determining both the rigid and nonrigid components of the mapping transformation. The mapping step is straightforward and involves labeling the point in the tCT that has the same prostate-relative coordinates as the planned isocenter using (2):

(2) $$\begin{array}{c} {\text{VAL}}_{\text{Mapped}}^{\prime }\left(M^{\prime }\left(i^{\prime },j^{\prime },k^{\prime }\right)\right)=\text{VAL}\left(M\left(i,j,k\right)\right), \end{array}$$

where  *M*,  *M*′ = prostate models in pCT and tCT, respectively, *i*, *j*, *k* and *i*′, *j*′, *k*′ are corresponding positions in *M*- and *M*′-relative coordinates, VAL = value of scalar, for example, label, dose, or intensity, in the pCT at *M*-relative position *i*, *j*, *k*,  and VAL_Mapped_′ = value  of  scalar mapped to *M*′-relative position *i*′, *j*′, *k*′.

### 2.4. Reference tCT Isocenters

The clinical and automapping protocols yielded two independent sets of tCT isocenters. To compare these sets, a reference isocenter was determined for each tCT by repeating the automapping procedure with human supervision. The main task was to reposition and/or edit the autosegmented prostate as necessary to achieve the best match with the tCT image data while generally preserving the global shape and volume defined by the planning contours. Every rigid registration and prostate segmentation were evaluated and edited based on human judgment, after which the tCT isocenter from (2) was accepted without modification. This procedure was performed without the pressure of clinical time constraints over ~8 months by two physicists and a dosimetrist working as a team. In general, the dosimetrist and one of the physicists made the initial pass, and the results from that pass were reviewed at a later time by the second physicist. The team met about once a week to discuss and review ongoing progress. Results from the dosimetrist/physicists team were periodically evaluated by one or two radiation oncologists based on the criterion of clinical reasonableness; that is, given the planning contours as truth, would the radiation oncologist judge the location and shape of the prostate in the tCT to be clinically reasonable? This criterion eliminated a potential source of interobserver bias and variability and effectively served the need for clinical standards. Only a few tCTs per patient were reviewed because of the large number of cases and the fact that the position and shape of the prostate in a given tCT are expected to be strongly correlated with other tCTs for the same patient.

### 2.5. Calculation of Hypothetical Delivered Dose Distributions

**Figure 6:**
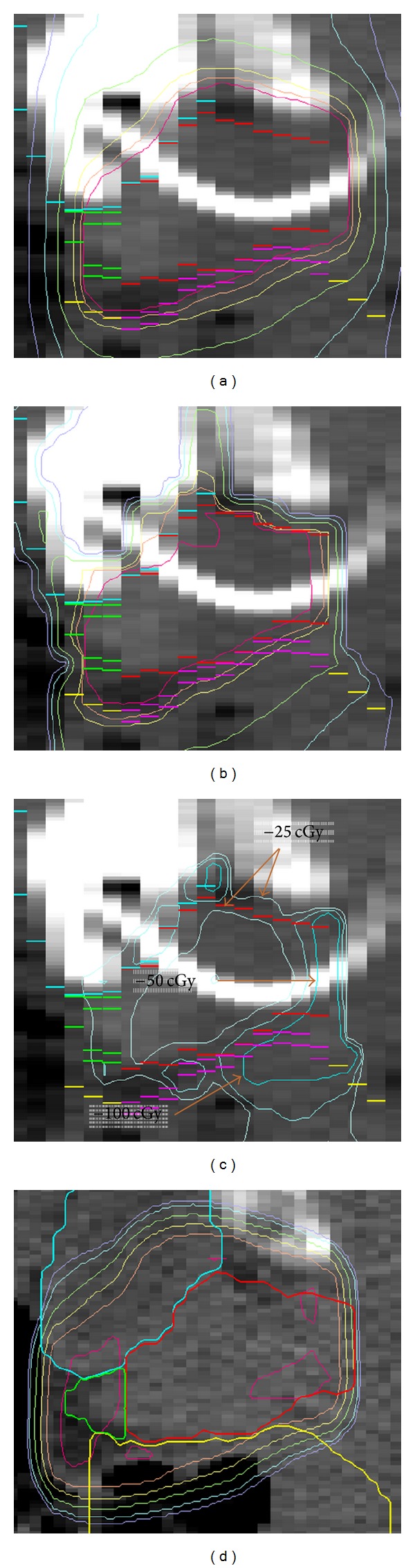
Organ contours and isodose curves on midsagittal slices for a typical case. Organs shown are the prostate (red), seminal vesicles (green), bladder (blue), rectum (yellow), and anterior rectal wall (purple). (a) Planned isodoses on pCT. (b) Cumulative isodoses on pCT. (c) Error dose on pCT. (d) Delivered isodoses computed from one tCT.

**Figure 7:**
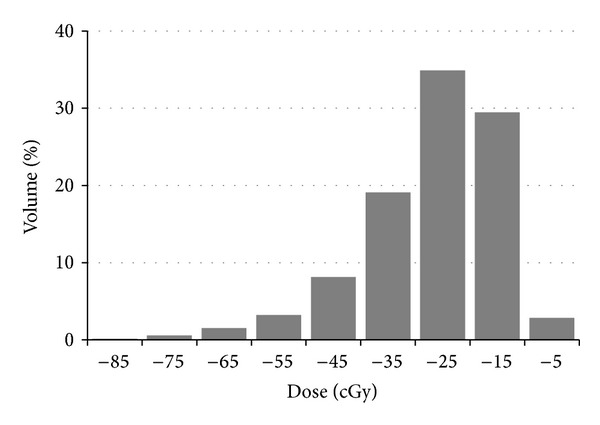
Prostate DVH for the error dose distribution shown in Figure 6(c). The bin size is 10 cGy.

Treatment dose distributions for each tCT and each mapped isocenter were computed using PlanUNC assuming all beams were delivered as planned for each of the three isocenters (Figure 6). This was accomplished by importing each tCT to PlanUNC, registering the ensemble of planned beams to each of the treatment isocenters in turn, and calculating the delivered dose assuming all beams were delivered as planned. After dose calculation the DICOM-RT files for the planned and treatment dose distributions were imported to ARTSuite and dose distributions were mapped from tCTs to the pCT using (2) in the reverse direction. Except for a rind ~2–5 mm thick around the prostate, dose to interstitial tissues was not mapped since (2) applies only to modeled tissues. Dose to the rind was mapped by extrapolating the skeletal framework a small distance beyond the prostate surface. The mapped treatment doses were summed over all tCTs and all isocenters and resampled to the grid of the planning dose to simplify comparison of planned and treatment dose distributions.  Figure 6(a) shows the planned isodose curves for the 10, 25, 50, 85, 90, and 95% levels on the pCT for the initial (nonboost) portion. The proximal two slices (6 mm length) of the seminal vesicles were included in the PTV.  Figure 6(b) shows the same isodose levels from the cumulative dose for the clinical protocol over 15 fractions. Error dose distributions (Figure 6(c)) were computed by subtracting the scaled planned dose distribution from the summed treatment dose distribution. The scaling factor was determined by the number of fractions contributing to the summed treatment dose. Isodose curves are shown for −100, −50, and −25 cGy.  Figure 6(d) shows the same isodose levels as in Figure 6(a) computed from one of the 15 tCTs. The frequency distribution in Figure 7 illustrates the expected underdosing of the prostate due to differences between prostate shape and location at planning and treatment times.

**Figure 8:**
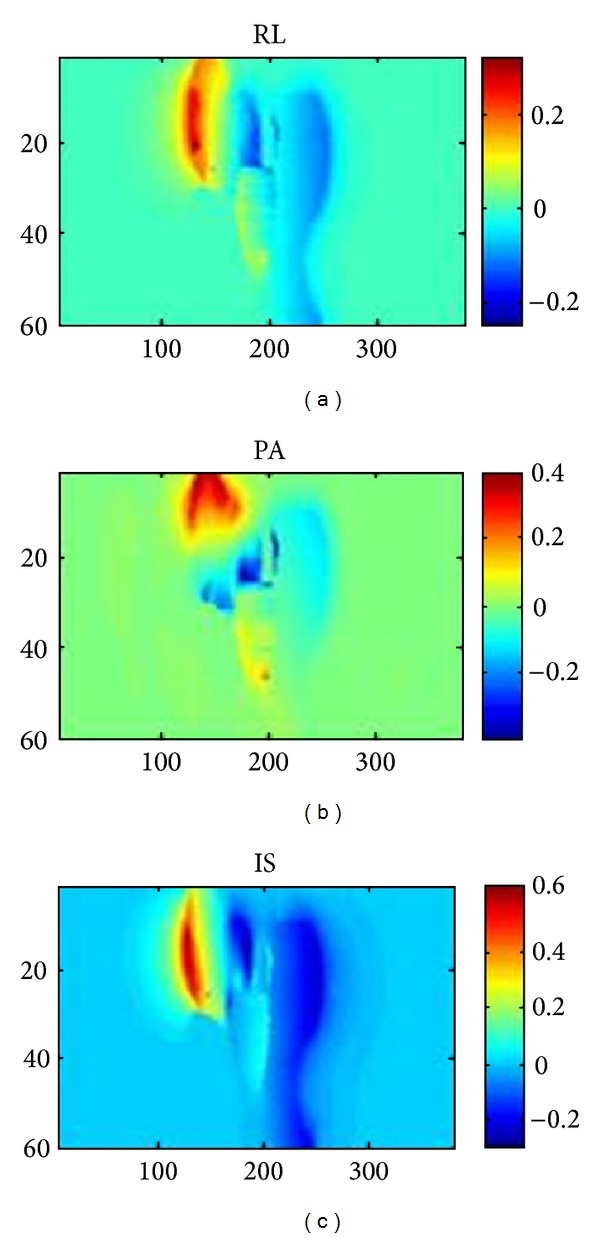
Color-coded difference maps for points in the tCT nonrigidly registered to the pCT using FEM and models. In the prostate ROI the agreement between the two methods is ~1 mm along the *x* and *y* axes and ~2 mm along the *z* axis. The larger difference along the *z* axis is attributed to the 3 mm slice thickness.

For dose accumulation purposes, the spatial accuracy required for nonrigid registration of a point in the tCT with its corresponding point in the pCT depends on the tolerable dose error and the steepness of dose gradients near the points. Assuming that the spacing between calculation points in the dose grid is matched to the dose gradients [19, 20], correspondence errors should be small compared to the grid spacing. The clinical planning grid spacing was 5 mm in this study.  Figure 8 shows color-coded maps of differences between the positions of two points, one point for each method, resulting from a single point in the tCT. The differences were ~1 mm along the *x* and *y* axes and ~2 mm along the *z* axis for the prostate ROI. The larger difference along the *z* axis is attributed to the 3 mm slice thickness. These findings support the use of m-reps for dose accumulation for this study. 

## 3. Results

### 3.1. Distance Metrics

**Figure 9:**
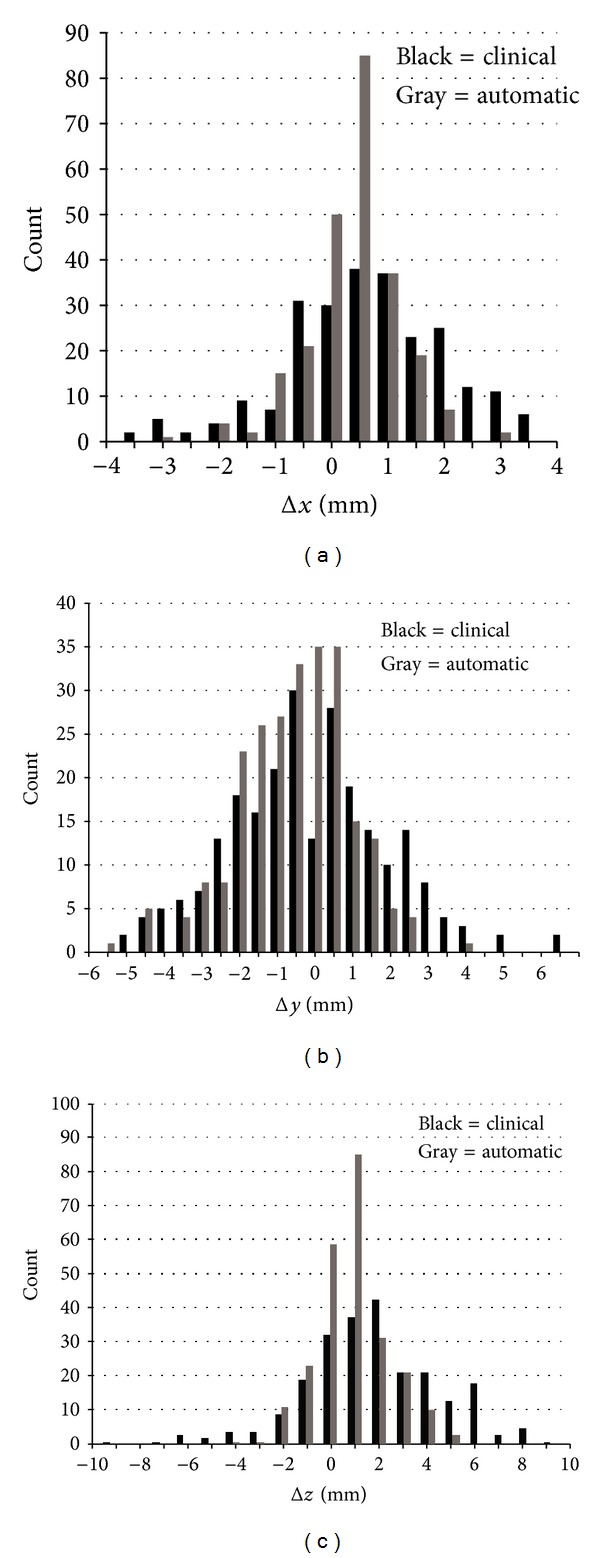
Frequency histograms for Δ*x*, Δ*y*, and Δ*z*. The averages of the unsigned clinical values for Δ*x*, Δ*y*, and Δ*z*, respectively, are 1.06 mm, 1.79 mm, and 2.54 mm. The averages of the automatic values are 0.58 mm, 1.14 mm, and 1.05 mm. Two clinical values are outside the Δ*y* axis range and seven values are outside the Δ*z* range. All of the automatic results are within the ranges of all axes.

**Figure 10:**
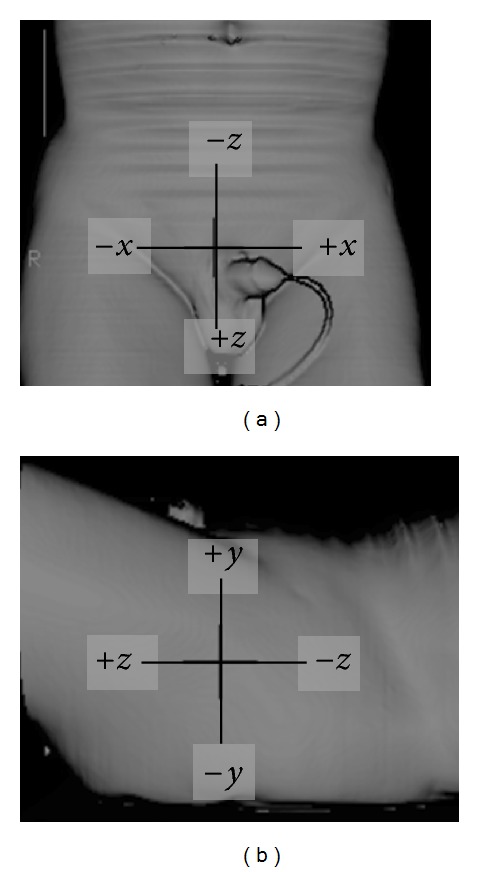
Coordinate system for calculating Δ*x*, Δ*y*, and Δ*z*.

Frequency histograms (Figure 9) were computed from the signed differences (Δ*x*, Δ*y*, Δ*z*) (Figure 10) between the reference isocenters and the clinical and automatic isocenters for all 243 tCTs. The bin width for Δ*x* and Δ*y* was 0.5 mm. To maintain comparable counts per bin, the width for Δ*z* was chosen to be 1.0 mm. Summary statistics for all ten patients are given in Table 2. Figure 9 shows that the distributions for clinical and automatic isocenters are centered near the reference values in approximately Gaussian fashion, supporting the utility of the reference values. 

Comparison of the frequency histograms for clinical and automatic protocols shows that automatic mapping is robust, as accurate as the clinical protocol along all three axes, and more precise than the clinical protocol, where accuracy is the average of the unsigned Δ*s* compared to the reference values, and precision is the spread (standard deviation) for each axis. Even though the averages for the automatic method are smaller (closer to the references) than the clinical protocol, greater accuracy is not claimed because the references are not golden. On the other hand, the standard deviation is characteristic of the registration method and independent of the reference values. The ANOVA *F*-test is <10^−12^ for all three axes, demonstrating that human and automatic variances are significantly different. As seen from Table 2, these observations apply for each individual patient. Moreover, the clinical protocol yielded 8 outliers, defined here as differences >3*σ*_clinical_ (*σ*_clinical_ = standard deviation of clinical protocol), with the largest being almost 5*σ*_clinical_. In comparison, the largest difference for the automatic approach is slightly less than 3*σ*_clinical_ along the *x* axis for patient 10, and for this case *σ*_clinical_ is small (*≈*1 mm). 

Table 2 also gives results for Court and Dong [12] and Smitsmans et al. [13]. Both studies evaluated automatic localization of the prostate in tCTs via multiscale rigid registration with pCTs. The values given in Table 2 for these studies are differences in prostate position, as opposed to isocenter position, between automatic methods and manually prepared references. Court and Dong reported results for two patients: patient A had 22 tCTs and was considered less challenging than patient B, who had 21 tCTs. Smitsmans et al. looked at a collection of 19 patients with 8–13 tCTs each. The results in Table 2 are for 91% of the tCTs. The remaining 9% were considered outliers and quantitative results were not reported. The automatic rigid registration method presented in this paper appears qualitatively to be comparable in performance to both Court and Dong and Smitsmans et al. No direct quantitative comparisons are possible however due to differences in study designs. 

### 3.2. Prostate DVHs

#### 3.2.1. Initial Portion of Treatment Regimen

**Figure 11:**
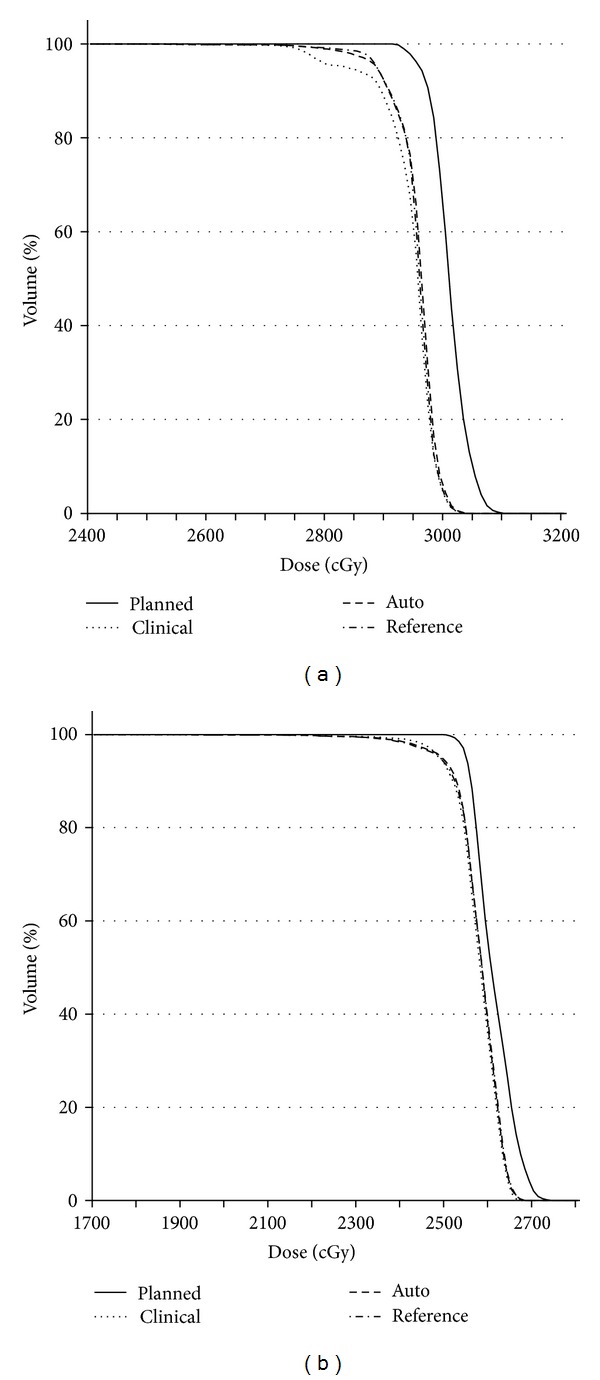
Prostate accumulated-dose DVHs for two patients. The planned doses have been scaled to the number of fractions accumulated for each patient.

Prostate DVHs for doses accumulated using the clinical, automatic, and reference isocenters for two typical patients are compared against the scaled planned DVHs in Figure 11. These cases illustrate (i) small differences among the three isocenters, (ii) degradation of the shoulder region, and (iii) a decrease in delivered versus planned dose of ~100–200 cGy scaled to the full nonboost portion. These general findings were consistent across all ten patients, but the severity of shoulder degradation was patient specific. Automatic mapping performed better in the shoulder region (Figure 11(a)) than the clinical protocol in about half the cases and as well in the other half (Figure 11(b)). The absence of more significant differences is attributed to margins (5 mm) that were relatively insensitive to isocenter mapping variations on the order of a few mm, a finding expected for properly designed margins.

Figure 7 shows the differential DVH for the error dose for the patient in Figure 11(a). Error isodose curves in Figure 6(c) show that the underdosed region occurs at the apex. The superior shift of the delivered dose distribution displayed on the tCT in Figure 6(d) was also present on other tCTs, explaining the origin of the under dose. Underdosing at the base was not observed for this patient because the planning target volume was enlarged superiorly to include the proximal SVs, providing extra protection at the base. However, underdosing was observed at both the base and apex for all patients whose SVs were not included in the target volume. 

#### 3.2.2. Boost Portion of Treatment Regimen

**Figure 12:**
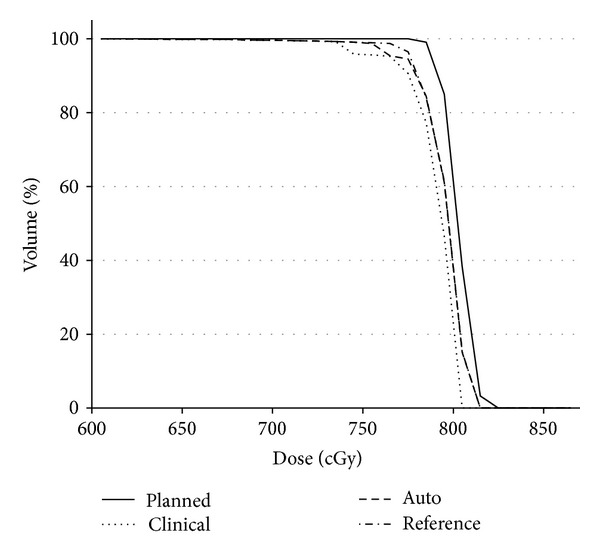
DVHs for a boost portion. The planned dose was scaled to four fractions.

TCTs for boost fractions were available for only two patients. DVHs in Figure 12 were computed from tCTs acquired for four of twelve boost fractions. The DVHs for both patients show the same general trends observed for the initial treatment portion (Figure 11). However, the clear separation between the descending portions of the clinical and automatic isocenters in Figure 12 suggests that automatic mapping may reduce the overall prostate under dose compared to the clinical protocol. If true, this finding would not be surprising since the boost margin (3 mm) would be expected to be more sensitive to image registration errors. However, further study is needed to test this finding.

## 4. Discussion and Conclusions

This work presents a formalism for mapping the planned isocenter to a tCT based on correspondence properties of a deformable prostate organ model that is used for registration of the pCT and tCT and for segmentation of the prostate in the tCT. The fully automatic mapping algorithm is as accurate as the clinical protocol but more precise. The algorithm had no failures or outliers for the tCTs studied. Better precision can be explained in terms of the robust properties of the algorithm and the absence of intra- and interuser variabilities. Moreover, human registrations were made under the pressure of clinical time constraints that can hasten decisions and lead to suboptimal results. 

In dosimetric comparisons for the prostate, automatic mapping showed less degradation in the shoulder region of DVHs for 50% of patients in this study. In the other 50%, degradation was no worse than the clinical protocol. The absence of large differences in dose-volume metrics is attributed to prostate margins that were relatively insensitive to variations in isocenter mapping on the order of a few mm. 

The clinical significance of the observed dosimetric improvements was not addressed but appears to be modest for the patients in this study. This conclusion however depends on the prostate margin and dose fractionation scheme as suggested by Figure 12. In particular, dosimetric improvements might be significant for less forgiving forms of treatment delivery such as stereotactic body radiotherapy. Also the finding that accurate pCT and tCT image registration does not fully compensate for geometric variability supports conclusions of other studies [2, 3] that full compensation for patient-specific geometric changes requires off-line adaptive planning or online reoptimization.

The overall conclusion is that the automatic algorithm robustly maps the planned isocenter to a position close to the correct location in a tCT and thus is well suited to augment human judgment in the clinical setting. Furthermore, the algorithm offers the potential for reducing registration outliers. During the workflow for mapping the isocenter, all of the essential image processing steps for calculation of delivered dose and mapping the delivered dose to planning space are performed. 

## Figures and Tables

**Table 1:** Isocenter Mapping Methods.

	Clinical mapping	Automatic mapping
1	CT simulate. Align patient with laser beams intersecting at simulated isocenter. Tattoo skin at centers of lateral and anterior laser beams.	Import pCT, structure sets, and isocenter to MxAnatomy. Fit models to planning contours.

2	Plan. Place crosshairs at planned isocenter in axial slice. Mark skin contour at lateral and anterior intersections with crosshairs.	Acquire tCT using standard procedures.

3	Prepare patient for treatment imaging. Tape BBs to anterior and lateral skin tattoos. Acquire tCT with laser beams centered on BBs.	Import tCT. Rigidly register pCT with pCT using automatic multiscale procedure.

4	Import tCT to PLanUNC. Autoregister imaged BBs in tCT with skin marks in pCT from Step 2.	Autosegment prostate in tCT.

5	Inspect registration by comparing prostate contours in pCT with intensity patterns in the tCT. Manually edit registration to get best match between contours and tCT intensities.	Determine correspondences between pCT and tCT prostate models.

6	Convert manual *x*, *y*, *z* edits from Step 5 to table shifts relative to laser beams. Apply shifts >3 mm and treat.	Map isocenter label from pCT to tCT via correspondence from Step 5 (2).

**Table 2:** Summary statistics over all patients.

Patient	Δ*X* (mm)						Δ*Y* (mm)						Δ*Z* (mm)					
	|Avg|^†^		Max, Min		Std Dev		|Avg|^†^		Max, Min		Std Dev		|Avg|^†^		Max, Min		Std Dev	
	Clinic	Auto	Clinic	Auto	Clinic	Auto	Clinic	Auto	Clinic	Auto	Clinic	Auto	Clinic	Auto	Clinic	Auto	Clinic	Auto
1	1.3	0.4	2.8, −3.0	1.7, −0.2	1.5	0.5	2.3	1.4	6.7, −6.0	0.2, −5.8	3.0	1.7	3.2	1.2	^¥^ **12.9**, −2.2	3.5, −1.4	4.1	1.4
2	1.1	0.4	3.1, −1.6	1.2, −0.7	1.3	0.5	1.4	0.9	4.8, −4.2	2.0, −2.4	1.8	1.1	1.9	0.6	6.4, −6.6	2.5, −2.2	2.7	0.9
3	1.1	0.5	1.7, −3.4	1.7, −0.9	1.4	0.6	1.9	0.9	1.8, −6.1	2.0, −4.0	2.2	1.3	1.7	1.0	2.7, −4.9	3.5, −2.1	2.1	1.3
4	1.0	0.6	2.8, −3.6	2.8, −1.3	1.4	0.8	1.7	1.0	^¥^ **11.7**, −6.8	0.5, −4.9	2.9	1.3	2.7	0.8	^¥^ **18.4**, −6.5	1.1, −3.1	4.2	1.1
5	0.7	0.6	2.4, −1.5	1.2, −1.2	0.9	0.7	1.4	0.9	2.9, −4.4	0.8, −4.7	1.7	1.2	2.3	1.5	^¥^ **11.2**, −2.2	4.5, −1.7	3.0	1.7
6	0.8	0.4	2.1, −1.6	0.6, −2.2	1.0	0.5	1.5	1.0	3.7, −4.3	2.5, −3.3	1.9	1.3	3.0	1.1	10.4, −4.8	3.3, −2.3	3.6	1.4
7	1.2	0.7	2.7, −3.5	0.2, −2.3	1.5	0.8	1.8	0.9	3.2, −5.2	2.1, −2.3	2.2	1.1	2.0	0.8	^¥^ **11.8**, −1.4	2.8, −4.7	2.7	1.2
8	0.9	0.3	3.2, −1.8	0.9, −1.2	1.2	0.3	1.4	0.7	3.3, ^¥^−**6.2**	0.8, −2.4	1.8	0.8	1.8	0.8	^¥^ **14.0**, −2.5	3.2, −1.9	2.8	1.1
9	0.9	0.6	3.3, 0.5	1.8, −0.4	1.0	0.6	1.4	0.8	3.0, −4.8	1.9, −1.3	1.9	0.9	2.9	0.8	4.4, −9.1	4.1, −1.3	3.6	1.1
10	0.7	0.6	2.9, −2.1	0.9, −3.1	1.0	0.8	1.0	0.8	4.6, −1.3	3.5, −2.0	1.3	1.2	2.0	1.3	5.3, −6.0	3.9, −1.6	2.6	1.5
All	1.1	0.6	3.3, −3.6	2.8, −3.1	1.4	0.8	1.8	1.1	11.7, −6.8	3.5, −5.8	2.3	1.5	2.5	1.1	^¥^ **18.4**, −9.1	4.5, −4.7	3.5	1.4
Reference [12] Pat A	NA*	0.0^#^		1.0^+^		0.5	NA*	0.2^#^		1.1^+^		0.5	NA*	0.3^#^		2.4^+^		1.0
Reference [12] Pat B	NA*	0.3^#^		1.4^+^		0.6	NA*	−0.2^#^		1.9^+^		1.4	NA*	−0.1^#^		3.5^+^		1.9
Reference [13]	NA*	0.1				0.7	NA*	0.1				1.2	NA*	0.0				1.3

^*¥*^Bold values are outliers. ^†^Average of unsigned differences. *Not applicable. ^+^Maximum. ^#^Average of signed differences.

## References

[1] Song WY, Schaly B, Bauman G, Battista JJ, van Dyk J (2006). Evaluation of image-guided radiation therapy (IGRT) technologies and their impact on the outcomes of hypofractionated prostate cancer treatments: a radiobiologic analysis. *International Journal of Radiation Oncology, Biology, Physics*.

[2] Yan D, Lockman D, Brabbins D, Tyburski L, Martinez A (2000). An off-line strategy for constructing a patient-specific planning target volume in adaptive treatment process for prostate cancer. *International Journal of Radiation Oncology Biology Physics*.

[3] Schulze D, Liang J, Yan D, Zhang T (2009). Comparison of various online IGRT strategies: the benefits of online treatment plan re-optimization. *Radiotherapy and Oncology*.

[4] Jannin P, Fitzpatrick JM, Hawkes DJ, Pennec X, Shahidi R, Vannier MW (2002). Validation of medical image processing in image-guided therapy. *IEEE Transactions on Medical Imaging*.

[5] Wong JR, Gao Z, Uematsu M (2008). Interfractional prostate shifts: review of 1870 computed tomography (CT) scans obtained during image-guided radiotherapy using CT-on-rails for the treatment of prostate cancer. *International Journal of Radiation Oncology, Biology, Physics*.

[6] Aird EGA, Conway J (2002). CT simulation for radiotherapy treatment planning. *British Journal of Radiology*.

[7] Sailer S, Chaney EL, Rosenman JG, Sherouse GW, Tepper JE (1992). Three dimensional treatment planning at the University of North Carolina at Chapel Hill. *Seminars in Radiation Oncology*.

[8] Chang SX, Cullip TJ, Rosenman JG, Halvorsen PH, Tepper JE (2002). Dose optimization via index-dose gradient minimization. *Medical Physics*.

[9] Pizer SM, Fletcher PT, Joshi S (2005). A method and software for segmentation of anatomic object ensembles by deformable m-reps. *Medical Physics*.

[10] Pizer SM, Broadhurst RE, Jeong JY, Frangi A, Delingette H Intra-patient anatomic statistical models for adaptive radiotherapy.

[11] Merck D, Tracton G, Saboo R (2008). Training models of anatomic shape variability. *Medical Physics*.

[12] Court LE, Dong L (2003). Automatic registration of the prostate for computed-tomography-guided radiotherapy. *Medical Physics*.

[13] Smitsmans M, Wolthaus J, Artignan X (2004). Automatic localization of the prostate for on-line or off-line image-guided radiotherapy. *International Journal of Radiation Oncology, Biology, Physics*.

[14] Maes F, Vandermeulen D, Suetens P (1999). Comparative evaluation of multiresolution optimization strategies for multimodality image registration by maximization of mutual information. *Medical Image Analysis*.

[15] Siddiqi K, Pizer S (2008). *Medial Representations: Mathematics, Algorithms and Applications*.

[16] Murphy MJ, Salguero FJ, Siebers JV, Staub D, Vaman C (2012). A method to estimate the effect of deformable image registration uncertainties on daily dose mapping. *Medical Physics*.

[17] Broadhurst RE, Stough J, Pizer SM, Chaney EL A statistical appearance model based on intensity quantiles.

[18] Frantz S, Rohr K, Stiehl H (2000). Localization of 3D anatomical point landmarks in 3D tomographic images using deformable models. Medical image computing and computer-assisted intervention. *Medical Image Computing and Computer-Assisted Intervention*.

[19] Niemierko A, Goitein M (1989). The influence of the size of the grid used for dose calculation on the accuracy of dose estimation. *Medical Physics*.

[20] Dempsey JF, Romeijn HE, Li JG, Low DA, Palta JR (2005). A Fourier analysis of the dose grid resolution required for accurate IMRT fluence map optimization. *Medical Physics*.

